# Religious life, monastic support, and healthcare inequity among aged Han Buddhist monks in China: an explanatory mixed-methods study

**DOI:** 10.3389/fpubh.2026.1911356

**Published:** 2026-10-01

**Authors:** Yaoping Liu, Zhenyu Liu, Chomphu Kotirum

**Affiliations:** Department of Global Buddhism, Institute of Science Innovation and Culture, Rajamangala University of Technology Krungthep, Bangkok, Thailand

**Keywords:** aged Han Buddhist monks, China, explanatory mixed-methods, healthcare access, healthcare inequity, monastic support, religious determinants, Traditional Chinese Medicine

## Abstract

This study examines the determinants of healthcare access inequity among aged Han Buddhist monks in China by focusing on the interaction between religious life, monastic support, and socioeconomic background. Using an explanatory mixed-methods design, the study first collected quantitative data from 170 respondents, including 70 aged Han Buddhist monks, 50 monastery administrators, and 50 health policy stakeholders. Multiple linear regression was used to examine the effects of religious factors, monastic/cultural support, and socioeconomic background on healthcare access. The quantitative results showed that religious factors negatively predicted healthcare access (*β* = −0.319, *p* = 0.001), whereas monastic/cultural support was the strongest positive predictor (*β* = 0.427, *p* < 0.001), followed by socioeconomic background (*β* = 0.131, *p* = 0.017). The model explained 50.7% of the variance in healthcare access. The qualitative phase, based on interviews with 20 participants, was then used to explain the mechanisms behind these statistical patterns. The findings show that preferences for Traditional Chinese Medicine, meditation, spiritual practices, and caution toward invasive procedures may delay engagement with biomedical care. Conversely, support from fellow monks, monastery administrators, donors, Buddhist associations, and government actors can expand access, particularly for monks without stable income or family-based support. Barriers were most pronounced in rural and independent monasteries, where transportation, insurance, internal health facilities, and policy integration remained limited. The study concludes that healthcare inequity among aged Han Buddhist monks cannot be reduced to economic or geographic disadvantage alone; rather, it is produced through the interaction of religious values, monastic support structures, and modern healthcare systems. Healthcare interventions for this population should therefore be monastery-based, culturally sensitive to Buddhist values, and integrated with public health policy.

## Introduction

Access to healthcare facilities among older people presents one of the most urgent issues related to modern health care systems because of its significance for patient safety, continuity of care, health care financing, and justice in delivering public services (1–3, 41, 44, 46). In general, definitions of access to healthcare cover not only facility availability but also opportunities of patients to receive affordable, accessible, timely, and appropriate services to meet their needs (4, 5, 37–40). For older patients, the process of receiving care depends on a number of factors including limited mobility, health literacy, burden of diseases, economic and social resources, and location close to healthcare facilities (6–8, 42).

Indeed, the problem is relevant in China due to the rapid aging of its population which puts a strain on the healthcare system, long-term care, and social security services. The Chinese population is made up of more than 264 million people aged 60 years and above, making up 18.70% of the total population (9). Research has shown that older people in China continue experiencing inequality in access to healthcare depending on place of residence, educational attainment, income, insurance, and urban vs. rural classification (10–13, 37–40). However, most of the studies analyze older people in terms of household, community, and urban vs. rural classification, whereas older people living in religious institutions have not been studied enough yet.

In case of aged Han Buddhist monks, there is an unusual cohort of aging people as they are placed in a different social position compared to common aged people living in families. Monks live in religious institutions, need less support from their biological families, do not have a steady income and usually rely on the support of monasteries, lay donations, Buddhist organizations, or religion-related networks for their basic needs and healthcare services. Thus, it is not possible to explain their access to healthcare in terms of common economic or geographical factors.

Religion can affect access to health care by shaping the views of aged monks concerning illness, the body, suffering, treatment, and care. Within Han Buddhism monasticism, illness can be understood not only from the biomedical point of view but also as spirituality, karmic knowledge, self-restraint, and acceptance of suffering (14, 15). Thus, religion can affect the willingness of monks to use biomedical treatment, delay formal treatment, and prefer non-invasive treatment options consistent with their spiritual beliefs (14, 15). Within the current research, Traditional Chinese Medicine (TCM), being a part of religious and cultural healthcare preferences (43, 47), will be considered one of the dimensions of religious factors. The use of TCM treatments, including herbal medicine, meditation, qigong practice, and spirituality, can be viewed as being more aligned with monastic values since it focuses on balance, moderation, and harmony of body and mind (16–18, 43, 47). Thus, the preference for TCM will not be considered a standalone variable but the dimension of religious-culture orientation, which can impact healthcare access (43, 47).

Another significant factor impacting access to healthcare is monastic support as many aged monks live in non-traditional families. Monasteries are not only places of religious life but also the social organizations, which provide support, information, help in organizing donations, connect the monk with different actors (such as lay community, Buddhist organizations, governmental bodies, healthcare organizations, etc.). From the point of view of the theory of social capital, relationships and institutional links between individuals and institutions can strengthen vulnerable people’s healthcare access opportunities (19). However, the monastic support may differ, depending on whether the monastery is urban or institutionalized and whether it is located in the city or in the rural area.

Previous research has focused on the relationship between religion and health in China including dimensions of religious beliefs, mental health, subjective wellbeing and spirituality in the Buddhist religion (17, 20, 21). Some research has been done on religious affiliations and healthcare usage in China (50). However, there has not been much attention paid to aged Han Buddhist monks as an institutionalized aged religious population, which has its healthcare access influenced by their religious beliefs, monastic system, socioeconomic conditions, and rural/independent monasteries differences. Consequently, little is known about the problem of healthcare inequality of old people living outside the usual family structure.

In order to fill this gap, the proposed study utilizes the explanatory sequential mixed methods approach. During the quantitative phase, the prediction will be made whether religious factors, monastic and cultural support, and socioeconomic condition impact aged Han Buddhist monk’s healthcare access. During the qualitative phase, the perception that relates to Traditional Chinese Medicine (TCM) will be analyzed in terms of quality and accessibility of health care for aged Han Buddhist monks and the reasons for rural or independent monasteries’ barriers to provide health services. Thus, healthcare access will be used as the outcome variable while healthcare usage, TCM preference, and quality of care as explanatory/interpretive variables.

Based on the above goals, three research questions are proposed in the current research. The first question is concerned with the influence of religious considerations, monk/culture-based support and socio-economic status on healthcare service access by aged Han Buddhist monks in China. The second research question is about the impact of monks’ attitudes toward Traditional Chinese Medicine versus biomedical healthcare on the quality and promptness of the service received. Finally, what barriers constrain healthcare support to aged Han Buddhist monks, especially in rural and autonomous monastic institutions?

## Methodology

This study used an explanatory mixed-methods design because healthcare inequity among aged Han Buddhist monks cannot be adequately explained by quantitative or qualitative data alone. The quantitative phase identified the relationships among religious factors, monastic/cultural support, socioeconomic background, and healthcare access, while the qualitative phase explained the mechanisms behind these statistical patterns. This design follows the logic of explanatory mixed-methods research, in which qualitative findings are used to deepen the interpretation of quantitative results (22, 23).

### Theoretical framework and hypotheses

This study adopts Andersen and Newman’s healthcare service utilization framework to explain healthcare access among aged Han Buddhist monks. In this framework, healthcare utilization is understood as health-related behavior shaped by individual characteristics, healthcare system factors, and the broader social environment (24). The framework is relevant because healthcare access among aged Han Buddhist monks is not determined only by personal health conditions, but also by religious orientation, monastic discipline, institutional support, socioeconomic position, and access to formal healthcare services. Accordingly, this study adapts the framework into three determinant groups: individual-religious determinants, healthcare system determinants, and social-monastic determinants. Individual-religious determinants include age, education, perceived health condition, length of stay in the monastery, Buddhist religious orientation, preference for Traditional Chinese Medicine (TCM), meditation, spiritual practice, and attitudes toward biomedical procedures. TCM preference is treated as part of a broader religious-cultural orientation because it is often embedded in Buddhist understandings of bodily discipline, balance, non-invasiveness, and spiritual harmony. Healthcare system determinants include the availability of medical facilities, insurance or financial assistance, distance to healthcare services, transportation access, affordability, and comfort in consulting biomedical professionals. Social-monastic determinants include support from fellow monks, monastery administrators, lay communities, donors, Buddhist associations, government actors, and healthcare-related institutions.

This adaptation is necessary because aged monks’ healthcare behavior cannot be understood as a purely individual decision. Unlike many older adults who rely primarily on family-based eldercare, aged Han Buddhist monks often live within institutional monastic settings where access to care is mediated by religious values, monastery capacity, community solidarity, donor support, and formal or informal links with public health systems. Therefore, healthcare access was positioned as the dependent variable in the quantitative phase, while religious factors, monastic/cultural support, and socioeconomic background were positioned as the main independent variables. Based on this framework, three hypotheses were tested:

*H1*: religious factors are negatively associated with healthcare access among aged Han Buddhist monks;

*H2*: monastic/cultural support is positively associated with healthcare access among aged Han Buddhist monks; and

*H3*: socioeconomic background is positively associated with healthcare access among aged Han Buddhist monks.

The qualitative phase was then used to explain the mechanisms underlying these relationships, particularly how religiously embedded treatment preferences shape engagement with biomedical care, how monastic support functions as an enabling resource, and why rural or independent monasteries experience stronger structural barriers to healthcare access.

#### Quantitative research

The quantitative stage was meant to discover patterns of healthcare access and to determine the extent to which religion, monastic/cultural support, and socioeconomic background may predict access to healthcare services among older Buddhist monks in Han monasteries. To ensure comparable results, a questionnaire was used to collect relevant data from the respondents living in different monasteries, having different statuses in the monastery and being stakeholders with varied backgrounds. Healthcare access became the dependent variable whereas religion, monastic/cultural support, and socioeconomic background became the primary independent variables. Religion was understood as the extent to which beliefs of Buddhism, spiritual practices, TCM preference, meditation, and attitude toward invasive biomedical procedures can affect decisions in relation to health care. In my research TCM preference is considered as a sub-dimension of religious-cultural factors due to its strong correlation with Buddhist understanding of discipline of the body, balance, non-invasiveness and spirituality. Monastic/cultural support is defined as assistance (emotional, logistical, financial and organizational) offered by other monks, monastic administrators, lay people, donors, Buddhist organizations, government agencies and healthcare institutions. Socioeconomic background consists of education, location of the monastery and time spent in the monastery. Multiple linear regression was applied to measure the contribution of the above mentioned predictors into healthcare access. The results obtained on the quantitative stage have become the basis for further qualitative research.

#### Operationalization and measurement of variables

The operationalization of healthcare access entailed the use of a composite index that used the Likert scale items measuring respondents’ perceptions regarding their access to healthcare services. This includes ease of access to healthcare facilities, presence of basic healthcare facilities within the monastery, financial barriers, distance barriers, coverage by insurance and financial help, ease of consulting health professionals, and knowledge about health policy. Because the above items were combined to form a composite score, it meant that healthcare access was a continuous dependent variable and therefore multiple linear regression analysis was used to examine it. The operationalization of the religious factors entailed items measuring the level of Buddhist beliefs, meditation and spirituality, preference for Traditional Chinese Medicine (TCM), religious limits for medical interventions, and attitude toward biomedical invasive treatment. The preference for Traditional Chinese Medicine (TCM) is a sub-dimension of religion-culture due to the fact that it is frequently related to bodily regulation, balance, non-invasive measures, spiritual harmony, and acceptance of suffering, rather than a mere preference for therapeutic purposes only within the context of Han Buddhism. Monastic/cultural support is operationalized through the items measuring support from other monks, monastery administration, lay community, donor organization, Buddhist organization, government organizations and organizations related to healthcare delivery. Socioeconomic status consists of education level, monastery location, and length of stay in the monastery.

In the current study, the education refers specifically to the formal secular education, which include elementary school, junior high school, senior high school, college education, and other forms of formal education. However, religious education, which may include study of scriptures, meditation practice, ethical conduct, doctrine learning, and lifelong religious education within the monasteries of Buddhist monks is not quantified. Therefore, formal education should be understood as a part of socioeconomic status in this study and not a complete measure of the learning capacity of the monks.

#### Instrument development and reliability

The questionnaire was developed based on Andersen and Newman’s (24) healthcare service utilization framework and relevant literature on healthcare access, aging, religious health practices, and social support. Because no single validated instrument directly captured healthcare access, religiously embedded treatment preference, and monastic support among aged Han Buddhist monks, the items were developed for this study and reviewed by experts in public health, sociology of religion, and Buddhist studies to ensure content relevance and cultural appropriateness. Healthcare access was measured as a self-reported composite index using Likert-scale items, not as actual utilization frequency. Internal consistency reliability was assessed using Cronbach’s alpha for the main subscales before regression analysis. The Cronbach’s alpha values indicated acceptable internal consistency for healthcare access (*α* = 0.81), religious factors (*α* = 0.86), and monastic/cultural support (*α* = 0.84).

#### Participants

Quantitative participants comprised 170 respondents representing three main actors in monastic community health access: 70 Han Buddhist monks, 50 monastery administrators, and 50 health policy stakeholders. Aged Han Buddhist monks were the main participants because they were the group who directly experienced obstacles, delays, choices, or negotiations in accessing health services. The monastery management is involved because they know the monastery’s internal mechanisms regarding health assistance, fundraising, transportation, daily maintenance, and relations with external parties. Health policy stakeholders were involved because they had information about policies, institutional support, the role of Buddhist associations, and the relationship between monasteries and the public health service system. Inclusion criteria for aged Han Buddhist monks were aged 60 years or older, living in a Han Buddhist monastery in Shandong or Zhejiang Province, having experience with access to health services, and willing to participate voluntarily. The inclusion criteria for monastery managers consisted of involvement in monastery administration as well as knowledge about health issues related to old monks in the monastery. The inclusion criteria for health policymakers included involvement in issues related to religion or health of monastics, actions taken by Buddhist groups, or policies concerning monastic health. Participants were excluded if they had severe cognitive impairment, serious communication limitations, or health conditions that made participation ethically inappropriate.

#### Sample size

For the quantitative sample, a total of 170 participants were selected, comprising 70 Han Buddhist monks, 50 management members of monasteries, and 50 health policy stakeholders. Such a number of samples is considered appropriate for exploratory regression since the research model uses very few predictor sets, namely religion, monastic help, and socio-economic status. The sample is also adequate for capturing variations in perspectives among actors experiencing health barriers, actors managing internal monastery support, and actors understanding external policies. The selection of participants, based on urban and rural monasteries in Shandong and Zhejiang Provinces, took into account different levels of accessibility, organizational capabilities, relations with the laity, and distance from official healthcare facilities. The present quantitative approach does not seek to provide an estimate of all Chinese Buddhist monks who belong to the ethnic group of Han Buddhism, but to reveal important patterns of behavior in the older religious population.

#### Quantitative sampling strategy

In the quantitative stage, a multistage sampling technique will be used. At the first stage, Shandong and Zhejiang Provinces were selected because of the high number of Han Buddhists in addition to the availability of different types of monasteries such as urban, rural, government-related, and autonomous monasteries. This will ensure that there is variation in terms of accessing healthcare services rather than comparing provinces.

In the second phase, the classification was done based on location and institution. Classification was made according to whether the monasteries were located in an urban or a rural area, while that of the institutions was made based on monasteries that had more institutional help than those without. It is important to make this classification since the access to healthcare services is influenced by the factors mentioned earlier.

In the third stage, respondents were selected from three groups: Han Buddhist monks, monastery managers, and health policy stakeholders. Aged monks were selected as the main group who directly experienced health access or barriers. The monastery administrators were chosen because they knew the internal support mechanisms. Health policy stakeholders were chosen because they understand the policy and external support. This gradual strategy was used because the population of older monks was not included in the general sampling list and had to be reached through the monastery’s institutional structure (25).

#### Quantitative data collection

Quantitative data were collected using a structured questionnaire from 170 respondents: 70 Han Buddhist monks, 50 monastery managers, and 50 health policy stakeholders. Questionnaires for aged monks are given in print and accompanied by researchers or field assistants. Questionnaires for monastery managers and health policy stakeholders are provided in print or online, depending on respondents’ access. All respondents received an explanation of the purpose of the research, the confidentiality of the data, and their right to refuse or terminate participation.

The questionnaire was prepared based on the healthcare service utilization framework from Andersen and Newman (24). The first part measures respondents’ characteristics, including age, education, perceived health condition, monastery location, and length of stay. Secondly, there is an evaluation of the ability to gain access to healthcare facilities including accessibility to the healthcare facilities, availability of basic health facilities in the monastery, constraints due to finances or distance, ease of talking to health experts, insurance coverage, and health policies awareness. The third section measures monastic support, including support from fellow monks, monastery administrators, lay communities, donors, Buddhist associations, and the government. The fourth section measures religious factors and treatment preferences, including preferences for TCM, the role of meditation and spiritual practices, limitations on medical procedures due to religious beliefs, and the tendency to avoid invasive biomedical procedures. All main items use a five-point Likert scale.

#### Quantitative data analysis

Quantitative Data Analysis Descriptive statistics and multiple regression analysis were used to analyze quantitative data. The use of descriptive statistics was to describe the demographics of respondents, access to healthcare facilities, monastic help, and religious factors. Multiple linear regression was used to test the influence of religious factors, monastic/cultural support, and socio-economic background on access to health services. Access to health services is positioned as the dependent variable. Religious factors, monastic/cultural support, education, the monastery’s location, and length of stay are positioned as predictors. Before interpreting the regression, tests for normality, linearity, multicollinearity, and homoscedasticity are conducted. The model is analyzed through the *R* value, *R* Square, Adjusted *R* Square, *F*, regression coefficient, standardized beta, *t* value, *p* value, and VIF. Quantitative results serve as the basis for the subsequent qualitative phase, according to the explanatory mixed-methods logic: the quantitative phase identifies patterns, while the qualitative phase explains the reasons and mechanisms behind those patterns (22, 23).

#### Qualitative research

After the quantitative phase showed that religious factors, monastic support, and socio-economic background were associated with aged Han Buddhist monks’ access to health services, a qualitative phase was conducted to explain the reasons behind this pattern. This phase focuses on three things: why religious factors can limit access to biomedical care, how monastic support strengthens access to health care, and which barriers are most important in rural or independent monasteries. Thus, qualitative research does not stand alone but serves to explain quantitative results according to the logic of explanatory mixed methods (22, 23).

#### Participants

Qualitative participants comprised 20 people: 10 Han Buddhist monks, 5 monastery managers, and 5 health policy stakeholders (Table 1). Aged monks were involved because they had firsthand experience of choosing, delaying, avoiding, or using health services. Monastery managers are involved because they know the forms of internal support, the limited facilities, the management of health funds, and the relationships with parishioners or donors. Health policy stakeholders are involved because they understand institutional support, health policy, and the monastery’s position in the public service system. The criteria for aged Han Buddhist monks follow those in the quantitative phase: being 60 years old or older, living in a Han Buddhist monastery in Shandong or Zhejiang, having experience in health services, and being willing to be interviewed. The monastery manager must have a role in managing the monastery and know the health arrangements of older monks. Health policy stakeholders must have involvement in religious affairs, Buddhist associations, health policy, or institutional support for monastic communities.

**Table 1:** Interview participants.

No	Initial name	Age	Role
1	EM01	72	Older Monk
2	EM02	69	Older Monk
3	EM03	74	Older Monk
4	EM04	66	Older Monk
5	EM05	71	Older Monk
6	EM06	68	Older Monk
7	EM07	70	Older Monk
8	EM08	73	Older Monk
9	EM09	67	Older Monk
10	EM10	75	Older Monk
11	MA01	51	Monastery Administrator
12	MA02	48	Monastery Administrator
13	MA03	55	Monastery Administrator
14	MA04	50	Monastery Administrator
15	MA05	52	Monastery Administrator
16	HS01	42	Health Policy Stakeholder
17	HS02	47	Health Policy Stakeholder
18	HS03	45	Health Policy Stakeholder
19	HS04	49	Health Policy Stakeholder
20	HS05	44	Health Policy Stakeholder

#### Sampling and recruitment strategy

The qualitative phase uses purposive and maximum variation sampling to obtain a heterogeneous sample of participants (26, 27). Participant variation was based on actor category, monastery location, monastery status, health access experience, and involvement in supporting the health of older monks. This strategy was used so that the interviews not only recorded the experiences of individual aged monks, but also captured the perspectives of monastery administrators and policy actors.

The selection of the older monks and monastery officials was made using an approach that involved working with the monastery officials who took part in the quantitative research stage. The participants of health policy were identified from the networks of the Buddhist associations, government agencies dealing with religious matters, or from health institutions for the monastic community.

#### Data collection

An interview guide was developed based on the findings of the quantitative phase. Findings regarding the negative influence of religious factors, the positive influence of monastic support, and the influence of socio-economic background on health access were used to develop interview questions. This approach follows the principle that interview guidelines in explanatory mixed-methods must bridge quantitative results with qualitative explanations (28).

Interviews were conducted in a semi-structured and in-depth manner. Questions for aged monks focused on experiences of accessing health services, reasons for choosing TCM or biomedical treatment, experiences of delaying treatment, cost barriers, distance, transportation, and the influence of Buddhist values on medical decisions. Questions for monastery administrators focused on internal facilities, financial assistance, community support, health records, and coordination with external parties. Questions for health policy stakeholders focused on policy, institutional support, administrative barriers, and the position of older monks in the health care system.

Interviews are conducted in person or via video call if face-to-face meetings are not possible. Each interview lasted approximately 45–60 min. Interviews were recorded with participant consent, transcribed verbatim, and coded anonymously. Data collection was stopped when the information obtained was repetitive and no longer produced new themes. This condition is considered an indication of data saturation (29). Data saturation was reached after the eighteenth interview, when no substantially new themes emerged. Two additional interviews were conducted to confirm thematic stability across participant groups.

#### Data analysis

Qualitative data were analyzed using deductive thematic analysis. Three dimensions in Andersen and Newman’s framework were used as initial themes, namely individual determinants, health system determinants, and social-monastic determinants. Analysis was carried out through reading transcripts, assigning initial codes, grouping codes into subthemes, and mapping subthemes into main themes (30).

Codes related to Buddhist beliefs, perception of pain, TCM preferences, meditation, and resistance to invasive procedures were included in the individual-religious determinants. Codes related to distance to services, costs, insurance, transportation, medical facilities, and access to biomedical care are included in the health system determinants. Codes related to support from fellow monks, monastery administrators, donors, laypeople, Buddhist associations, and the government are included among the socio-monastic determinants.

The results of qualitative analysis are used to explain quantitative results. Interview data is not only presented as supporting quotes but also used to explain the mechanisms by which religious factors can weaken access to biomedical care, why monastic support can strengthen access, and why rural or independent monasteries face greater obstacles.

#### Ethical considerations

The current research obtained approval from the Naresuan University Network Research Ethics Committee, Phitsanulok, Thailand under the Expedited Review process. The ethical approval number provided by the ethics committee for the research is NRE No. P4-0017/2568; COA No. 009/2025. The approval is valid from 21 November 2025 up to 21 November 2026. The research has been approved for conduct as per the guidelines that ensure proper protection of human subjects as mentioned in the Declaration of Helsinki, Belmont Report, CIOMS Guideline, and ICH-GCP.

All participants received information about the purpose of the study, data collection procedures, the confidentiality of their identities, the right to refuse participation, and the right to terminate participation at any time without consequences. Participant consent was obtained before completing the questionnaire; interviews and observations were carried out. Participants’ identities were disguised using codes such as EM for aged Han Buddhist monks, MA for monastery administrators, and HS for health policy stakeholders. Research data is only used for academic purposes and is reported without disclosing personal identity or sensitive institutional information.

## Result

### Quantitative research

#### Respondent characteristics

A total of 170 respondents participated in the quantitative phase, comprising 70 Han Buddhist monks aged 60 and above, 50 monastery managers, and 50 health policy stakeholders (Table 2). As shown in Table 1, respondents were predominantly aged over 70 years (38.8%), followed by those aged 66–70 years (32.9%) and 60–65 years (28.2%). In terms of education, the majority of respondents were at junior high school and senior high school level (51.7%), while 8.8% had no formal education. Most respondents’ health conditions were in the average (37.6%) and good (34.1%) categories, while 17.1% reported poor to very bad conditions. As many as 57.1% of respondents had lived in monasteries for more than 10 years. By location, 54.1% of respondents were from urban monasteries and 45.9% from rural monasteries. This composition shows that the quantitative data include variations in age, education, health status, length of stay in the monastery, and monastery location.

**Table 2:** Quantitative respondent characteristics (*N* = 170).

Characteristics	Category	*N*	Percentage (%)
Age (year)	60–65	48	28.2%
	66–70	56	32.9%
	>70	66	38.8%
Education	No education	15	8.8%
	Elementary school	36	21.2%
	Junior high school	41	24.1%
	Senior high school	47	27.6%
	Bachelor degree	21	12.4%
	Others	10	5.9%
Current health condition	Very good	19	11.2%
	Good	58	34.1%
	Average	64	37.6%
	Poor	21	12.4%
	Very bad	8	4.7%
Long stay in the monastery	<5 years	22	12.9%
	5–10 years	51	30.0%
	>10 years	97	57.1%
Monastery location	Urban	92	54.1%
	Rural	78	45.9%

It should be noted that the education categories in Table 2 refer to formal secular schooling. They do not capture Buddhist monastic education, scriptural training, meditation discipline, ethical reasoning, or lifelong religious learning. Therefore, the reported education distribution should not be interpreted as a complete indicator of monks’ overall educational experience or their capacity for health-related reasoning.

#### Health care access patterns, monastic support, and religious factors

As shown in Figure 1, the quantitative patterns show three main trends. First, access to health services is at a moderate to quite good level, but not evenly distributed. Respondents found it relatively easy to access facilities outside the monastery and felt comfortable consulting with medical personnel, but still faced barriers related to cost, insurance, location, and limited policy information. Second, monastic support is the strongest dimension. The support of fellow monks, monastery management, the community of believers, and external parties acts as a social resource that helps monks face the limitations of formal services. Third, religious factors have an ambivalent position. Meditation and spiritual practices are considered important for health, but a preference for TCM and a tendency to avoid invasive biomedical procedures may delay access to formal medical services.

**Figure 1:**
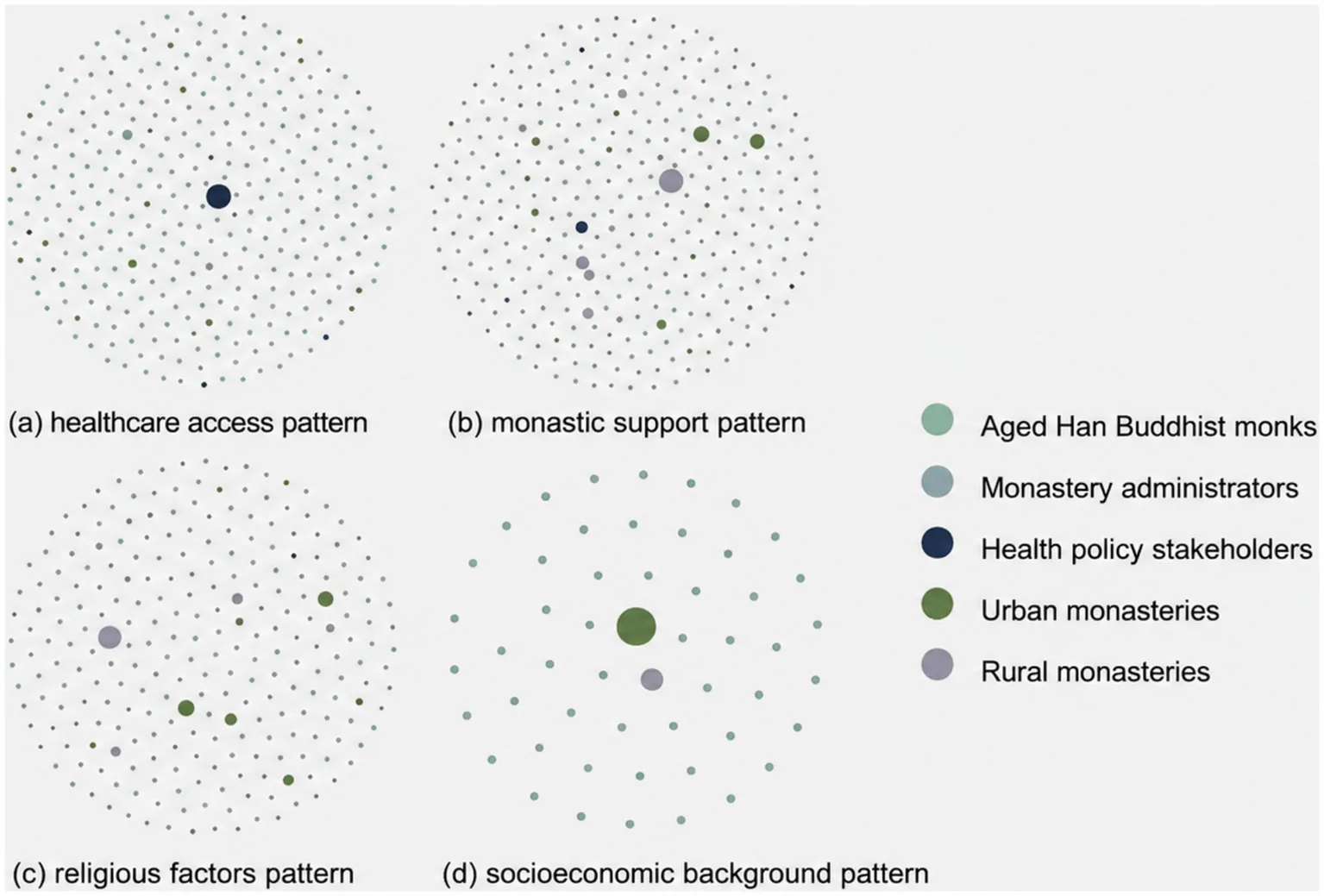
Quantitative patterns of healthcare access, monastic support, religious factors, and socio-economic background among 170 respondents (colors distinguish respondent groups and monastery locations).

As can be observed in Figure 1, access to health care for aged Han Buddhists is not a unidirectional issue, rather it is characterized by several dimensions including accessibility, monastic support, religion, and socio-economic conditions. This distribution implies that barriers to health care are partly determined by monastic and non-monastic factors, while on the other hand, religion can contribute to spiritual strength and limit medical help at the same time. This pattern provides the empirical basis for the regression analysis that follows, which tests the extent to which religious factors, monastic/cultural support, and socio-economic background predict healthcare access.

#### Multiple linear regression

Multiple linear regression was used to test the influence of religious factors, monastic/cultural support, and socio-economic background on access to health services. As shown in Table 3, the regression model was statistically significant, *F*(5, 111) = 22.835, *p* < 0.001. The model explained 50.7% of the variation in health care access (*R*^2^ = 0.507; Adjusted *R*^2^ = 0.491). These results indicate that the combination of religious factors, monastic support, and socio-economic background has a strong apparent influence on access to health care for aged Han Buddhist monks.

**Table 3:** Multiple linear regression of factors influencing access to health services.

Predictor	*B*	SE	*β*	*t*	*p*
Constant	8.371	2.414	–	3.468	0.001
Religious factors	−0.482	0.138	−0.319	−3.493	0.001
Monastic/cultural support	0.611	0.115	0.427	5.313	<0.001
Socioeconomic background	0.618	0.321	0.131	1.925	0.017

*R* = 0.712; *R*^2^ = 0.507; Adjusted *R*^2^ = 0.491; *F* (5, 111) = 22.835; *p* < 0.001.

Religious factors have a significant negative effect on access to health services (*β* = −0.319; *p* = 0.001). That is, the stronger the religious inclination that drives monks to choose TCM or spiritual practices, or to avoid biomedical procedures, the lower their access to formal health services. On the other hand, monastic/cultural support has the strongest positive effect on access to health services (*β* = 0.427; *p* < 0.001). This means that support from fellow monks, monastery administrators, donors, Buddhist associations, and external actors strengthens aged monks’ ability to access health services. Socio-economic background also has a positive and significant effect (*β* = 0.131; *p* = 0.017), indicating that education, the monastery’s location, and length of stay in the monastery also determine access to health.

Based on these results, all three hypotheses were supported. H1 was supported because religious factors showed a significant negative association with healthcare access (*β* = −0.319, *p* = 0.001). H2 was supported because monastic/cultural support showed a significant positive association with healthcare access and emerged as the strongest predictor in the model (*β* = 0.427, *p* < 0.001). H3 was also supported because socioeconomic background showed a significant positive association with healthcare access (*β* = 0.131, *p* = 0.017). These findings indicate that healthcare access among aged Han Buddhist monks is shaped by the combined influence of religious orientation, enabling monastic resources, and socioeconomic conditions.

#### Model of determinants of health care access

As shown in Figure 2, the regression results show that aged Han Buddhist monks’ access to health services is shaped by three main pathways. The first pathway is the religious pathway, which acts as a limiting factor when spiritual preferences, TCM, and resistance to invasive procedures reduce the likelihood of accessing biomedical care. The second path is the monastic path, which works as a strengthening factor through the support of the community, monastery administrators, donors, and religious institutions. The third path is the socio-economic path, which operates through education, the monastery’s location, and the experience of living in a monastic community. Such a trend highlights that health care disparity among aging Han Buddhist monks is not just due to accessibility to medical services but due to the interrelationship between religion, the support system, and socio-economic status.

**Figure 2:**
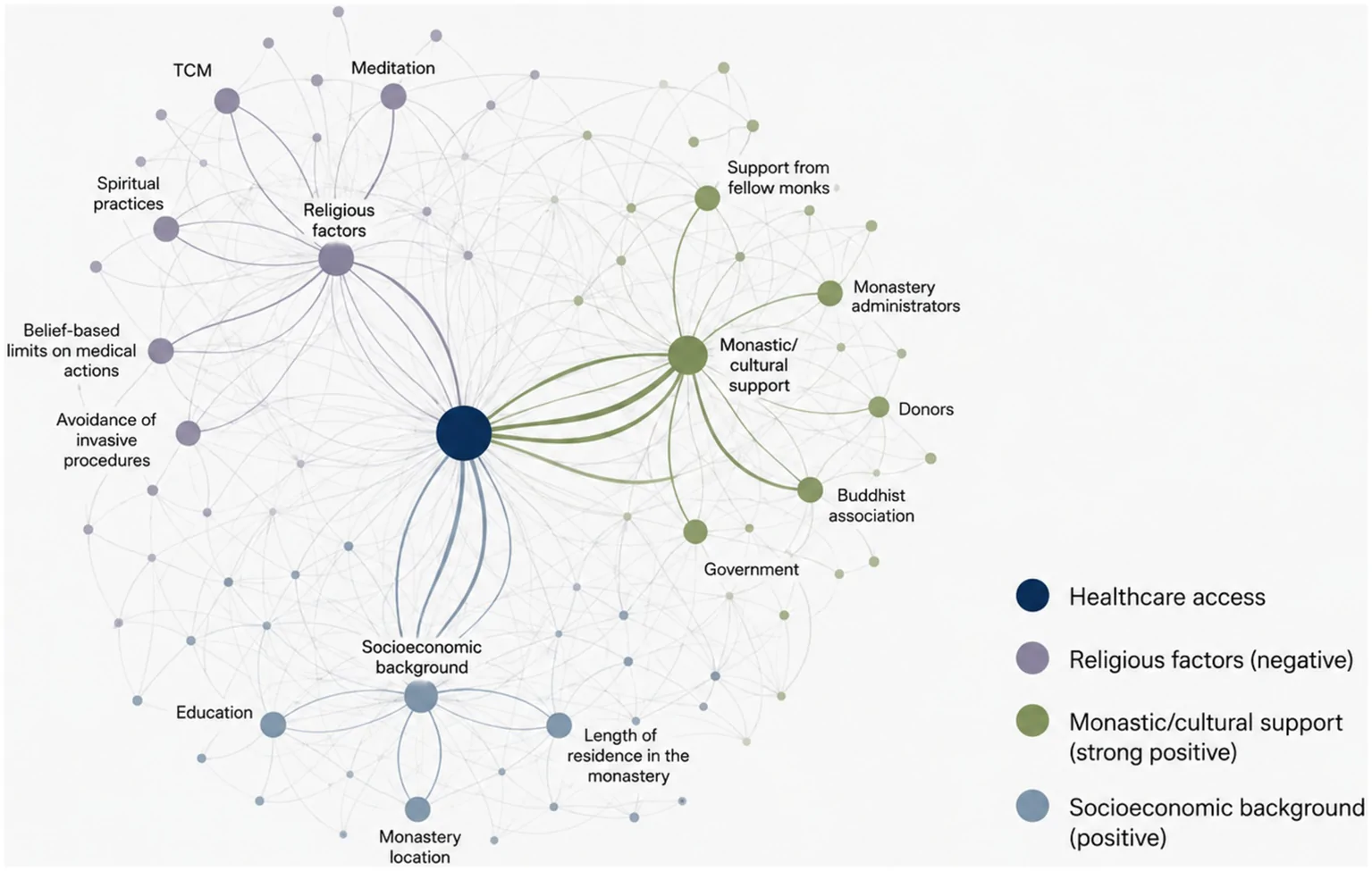
Quantitative model of determinants of healthcare access among aged Han Buddhist monks (the central node represents healthcare access).

Figure 2 emphasizes that the quantitative results not only show relationships between variables but also identify initial mechanisms that require further explanation. The distinct directions of influence among religious factors, monastic/cultural support, and socio-economic background indicate that a combination of value constraints, community capacity, and structural position shapes older monks’ access to health care. Therefore, this model provides the analytical basis for a qualitative phase to explore why religious factors limit the use of biomedical care, how monasteries support work as a health resource, and why rural or independent monasteries continue to face stronger access barriers.

The quantitative findings primarily answered the first research question by identifying the direction and strength of the effects of religious factors, monastic/cultural support, and socioeconomic background on healthcare access. However, the quantitative phase could not fully explain how TCM-oriented perceptions shaped the perceived quality and timeliness of care or why rural and independent monasteries faced specific barriers to support. Therefore, the qualitative phase was used to deepen the interpretation of RQ2 and RQ3 and to explain the mechanisms underlying the statistical patterns identified in RQ1.

### Qualitative research

The qualitative phase was carried out to explain quantitative patterns that show that religious factors, monastic/cultural support, and socio-economic background influence access to health services for aged Han Buddhist monks. Three main themes and eight subthemes were identified from interviews with aged monks, monastery administrators, and health policy stakeholders. Characteristics of interview participants are presented in Table 1, while a summary of themes, subthemes, and participant evidence is presented in Table 4.

**Table 4:** Summary of themes, subthemes, and participant evidence from the qualitative study.

Themes	Sub-themes	Participant evidence
Religious determinants	TCM-oriented health perception	EM01 and EM05 reported that herbal medicine, meditation, and spiritual practice were more consistent with monastic life than direct biomedical intervention. EM06 emphasized that treatment should not disturb inner peace or bodily discipline.
Religious determinants	Belief-based limits on biomedical treatment	EM03 and EM08 described illness as something that should be endured with awareness. EM06 showed reluctance toward invasive medical procedures. MA02 noted that some monks delayed formal treatment because medical intervention was viewed as spiritually uncomfortable.
Monastic and cultural support	Internal support from the monastery	MA01 and MA03 explained that monastery administrators often arranged transport, basic care, and financial support when aged monks needed treatment. EM07 stated that fellow monks became the first source of help before external medical services were contacted.
Monastic and cultural support	External institutional support	HS01 and HS03 stated that Buddhist associations, donors, and local government actors could improve access, but support remained uneven across monasteries. MA05 reported that small monasteries depended heavily on irregular donations.
Structural and socioeconomic barriers	Geographic and transportation barriers	EM01, EM05, and EM10 reported that distance from hospitals and lack of reliable transport delayed medical visits. Observation data also indicated that access to hospitals from rural monasteries required external assistance.
Structural and socioeconomic barriers	Financial and insurance constraints	EM03 and EM07 reported that treatment costs were a major barrier. MA05 explained that medical support was often available only when donations were sufficient.
Structural and socioeconomic barriers	Limited internal health infrastructure	MA01 and MA03 reported that most monasteries had no permanent medical staff, routine health records, or formal referral system. Observation data showed that health facilities inside monasteries were limited and mainly informal.

### Religious determinants

#### TCM-oriented health perception

Most aged monks interpret health through a spiritual and traditional framework. TCM, herbal remedies, meditation, and spiritual practices are not presented as additional options but as forms of treatment considered more in tune with monastic life. Some monks view traditional medicine as more in line with the principles of simplicity, body–mind balance, and acceptance of suffering. This pattern helps explain why religious factors were negatively associated with healthcare access in the quantitative results. The association does not necessarily indicate a complete inability to access biomedical services; rather, it reflects the possibility that some monks intentionally prioritized TCM, meditation, or spiritual practices before seeking biomedical diagnosis or treatment.

#### Belief-based limits on biomedical treatment

Religious values also form limits on certain medical procedures. Some aged monks show caution toward invasive procedures because they are considered to disturb inner peace, bodily discipline, or the principle of not harming the body. In some cases, pain is understood as part of spiritual practice or a karmic consequence that must be accepted with awareness. This view does not necessarily indicate rejection of biomedical care. Rather, it shows that some monks actively sequence their treatment decisions by first relying on spiritual practice, TCM, or non-invasive care before seeking biomedical services when symptoms become more severe or when monastic support enables referral. Monastery administrators and health policy stakeholders consider that this obstacle cannot be overcome simply by regular medical education but requires an approach sensitive to Buddhist beliefs.

### Monastic and cultural support

#### Internal support from the monastery

The monastery’s internal support is the primary resource for accessing healthcare for aged monks. This support includes caring for fellow monks, assistance from monastery management, transportation arrangements, fundraising, and assistance with accessing medical facilities. These findings explain why monastic/cultural support is the strongest positive predictor of quantitative outcomes. In the context of older monks who do not necessarily have biological family support or a steady income, monasteries serve as a social protection network that replaces some functions of the family and the formal welfare system.

#### External institutional support

Apart from internal support, monks’ access to health is also influenced by external support from believers, donors, Buddhist associations, local governments, and policy actors. However, this support is not evenly distributed. Monasteries that are larger, urban, or have institutional connections tend to have an easier time obtaining medical assistance, donations, or coordination with health facilities. In contrast, rural or independent monasteries depended more on unstable, informal aid. Health policy stakeholders emphasize that there is not yet a sufficiently systematic mechanism to ensure that aged monks in small and rural monasteries receive sustainable health support.

### Structural and socio-economic barriers

#### Geographic and transportation barriers

Geographical barriers are a major problem for rural or independent monasteries. Long distances to hospitals, limited transportation, road conditions, and dependence on others mean that access to health services is not always available when needed. Some aged monks postpone examinations because traveling to a medical facility requires money, time, and external assistance. These barriers suggest that monastery location is not simply a demographic variable, but a structural factor that determines when and how monks can access health services.

#### Financial and insurance constraints

Cost and insurance limitations also restrict access to health care. Many older monks lack a steady income and rely on monasteries, donations from parishioners, or community aid. The costs of consultations, medications, transportation, and follow-up care are often cited as reasons for delaying treatment. Monastery administrators stated that the monastery’s ability to help with health costs is highly dependent on the amount of donations and the economic capacity of the supporting community. This condition explains why socio-economic background has a positive effect on health access in quantitative results.

#### Limited internal health infrastructure

Some monasteries, especially rural or independent ones, lack adequate internal healthcare facilities. There are no permanent medical personnel, health recording systems, routine examinations, or clear referral procedures. Treatment is often carried out reactively, that is, when monks are already sick or need emergency help. These limitations make promotive and preventive efforts difficult to carry out. For health policy stakeholders, the absence of an internal monastery health system indicates that aged monks remain poorly integrated into formal health services for aged people.

#### Administrative and policy gaps

The final obstacle arises at the policy and administrative level. Some aged monks are not fully connected to insurance schemes, social assistance, or the public health system. Monasteries are also not always treated as social units requiring specialized older health interventions. Health policy stakeholders note that older monks are less visible in health policy because they do not fully fit into the categories of ordinary households, formal workers, or recipients of family support. Thus, healthcare inequality among aging Han Buddhist monks reflects the gap between modern healthcare structures and the character of monastic life.

## Discussion

This study provides empirical evidence on how religious life, monastic support, and socio-economic background shape healthcare inequity among aged Han Buddhist monks in contemporary China. The findings show that healthcare access in this population is determined not only by the availability of medical facilities but also by the interaction among religious orientation, monastic support structures, and structural socio-economic conditions. The regression results indicate three distinct pathways. Religious factors negatively predicted healthcare access; monastic/cultural support positively predicted access, with the strongest effect; and socio-economic background also contributed positively. These findings extend existing studies on healthcare-seeking behavior among older adults in China, which have emphasized education, residence, insurance, health status, and urban–rural disparities as key predictors of service use (31, 32, 45). The present study adds that for aging religious populations, healthcare inequity cannot be understood without examining spiritual beliefs, monastic life, and institutional support within religious communities.

The regression findings should be interpreted with caution. The *R*^2^ value indicates the explanatory power of the overall model, not the independent contribution of religious factors alone. Therefore, the findings do not suggest that religiosity by itself determines healthcare access. Rather, they show that healthcare access among aged Han Buddhist monks emerges through the combined influence of religious-cultural orientation, enabling monastic resources, and socioeconomic conditions. Although the regression model identifies significant statistical associations, these relationships should not be read as strictly causal or isolated pathways. Religious beliefs, monastic support, socioeconomic position, and healthcare access are likely reciprocal and interdependent. For example, religiously shaped treatment preferences may influence when monks seek biomedical care, while monastic support may reduce structural barriers and shape how monks interpret available healthcare options. Similarly, rural or independent monastery status may weaken access to formal services while increasing reliance on internal monastic and donor-based support. Thus, the model should be understood as an analytical representation of interacting pathways rather than as a set of independent causal mechanisms.

A key conceptual contribution of this study is its intersectional understanding of vulnerability. Healthcare inequity among aged Han Buddhist monks does not arise from religion alone, aging alone, or socioeconomic disadvantage alone. Rather, it emerges from the intersection of advanced age, monastic identity, institutional dependence, rural or independent monastery location, and limited reliance on conventional family-based eldercare. These intersecting positions may compound risk in several ways: advanced age increases health needs and mobility limitations; monastic identity shapes treatment preferences and moral interpretations of illness; institutional dependence makes access contingent on monastery capacity; rural location increases distance and transport barriers; and the absence of family-based support makes monks more dependent on monastic and donor-based assistance. At the same time, these intersections may also produce resilience. Monastic discipline, spiritual practice, fellow-monk solidarity, lay donations, Buddhist associations, and monastery-based networks can function as social capital that partially compensates for weak family support or limited personal income. Therefore, aged monks should be understood neither simply as economically disadvantaged older adults nor as passive victims of exclusion, but as religious aging actors whose vulnerability and resilience are jointly produced through the interaction of religious, institutional, geographic, and social positions.

The first major finding is that religious factors served as a constraining pathway to access biomedical healthcare. Aged monks who strongly preferred TCM, meditation, spiritual practice, and non-invasive care were less likely to engage actively with formal biomedical services. This pattern does not mean that religious life is inherently opposed to health; rather, it shows that a religiously grounded perception of health can redefine what counts as appropriate care. For many aged monks, TCM and meditation are not merely therapeutic alternatives but morally and spiritually congruent practices. Biomedical treatment, especially invasive procedures, may be perceived as disruptive to bodily discipline, inner peace, or Buddhist understandings of suffering. This finding is consistent with recent work showing that religious beliefs can transform health concepts and shape healthcare decision-making in China (33), as well as studies on Buddhist temple soundscapes and spiritual practices that link Buddhist environments with mental health and wellbeing (17). However, the present study goes further by showing that religious congruence may also delay diagnosis, reduce preventive care, and weaken engagement with biomedical systems when spiritual care becomes the dominant pathway.

It is therefore important to distinguish healthcare access, healthcare utilization, and healthcare preference when interpreting the negative coefficient for religious factors. In this study, healthcare access refers to monks’ perceived ability to obtain services that are reachable, affordable, acceptable, and institutionally supported; healthcare utilization refers to the actual use of biomedical or other healthcare services; and healthcare preference refers to culturally and religiously informed choices regarding TCM, meditation, spiritual practice, non-invasive care, or biomedical treatment. The negative association between religious factors and healthcare access should not be interpreted simply as physical inability to reach healthcare facilities or as passive exclusion from the health system. Rather, it reflects a more complex pattern in which some aged monks may have access to biomedical services but intentionally prioritize TCM or spiritual practices because these modalities are perceived as more consistent with Buddhist moral reasoning, bodily discipline, and understandings of suffering.

The qualitative findings further show that aged monks exercise agency within constrained structural environments. Their preference for TCM, meditation, spiritual practice, or non-invasive care should not automatically be interpreted as a barrier to biomedical treatment, but as a morally situated choice through which they actively negotiate illness, suffering, and appropriate care. In practice, monks may combine spiritual practice, traditional healing, biomedical consultation, and available monastic support according to what they consider religiously appropriate, morally acceptable, and practically possible. However, this agency is not exercised in ideal conditions. It operates within constraints such as rural location, limited transportation, unstable financial resources, weak insurance integration, lack of internal health infrastructure, and uneven institutional support. Therefore, healthcare decision-making among aged Han Buddhist monks should be understood as a negotiation between Buddhist moral agency and structural vulnerability, rather than as simple non-compliance with biomedical care.

The second major contribution of this study is the identification of monastic/cultural support as the strongest positive determinant of healthcare access. Support from fellow monks, monastery administrators, donors, Buddhist associations, and government actors increased the ability of older monks to access healthcare despite limited personal income, weak family support, or distance from formal healthcare facilities. This finding suggests that monasteries function as informal welfare infrastructures. They do not merely provide spiritual shelter; they also mediate access to transport, financial assistance, information, emotional support, and external institutional networks. This extends the social support literature by showing that, in monastic settings, community support operates as a substitute for family-based eldercare and as a bridge to public healthcare systems. It also aligns with recent studies emphasizing that older adults’ healthcare access depends not only on individual capacity but also on community-level resources, institutional mediation, and care infrastructures (34). Therefore, health interventions for aged monks should not target individuals alone, but should strengthen monasteries as community-based health access points.

The qualitative findings further clarify why monastic support matters. In large or urban monasteries, administrators and external networks could mobilize transportation, donors, and medical contacts more effectively. In rural or independent monasteries, support was weaker, less predictable, and more dependent on informal donations. This explains why socio-economic background and monastery location remained important in the quantitative model. The problem is not only that rural monasteries are geographically distant from hospitals. The deeper problem is that rural and independent monasteries often lack stable health records, routine screening, formal referral pathways, insurance integration, and permanent medical partnerships. These results resonate with current literature regarding barriers to rural healthcare in terms of geographic distance, transportation problems, limitations in services, and resource disparities, all of which are associated with geography (35, 36, 48, 49). In our current study, the above barriers are exacerbated by the social class of the monk population, whose members usually do not live within normal family-based eldercare settings.

This study also identified the limitations of relying only on regression to explain healthcare access. Regression was useful for identifying the direction and strength of relationships between religious factors, monastic/cultural support, socio-economic background, and healthcare access. However, it could not fully explain why religious beliefs discouraged biomedical engagement, how monastery support actually operated, or why rural and independent monasteries faced deeper barriers. This limitation is consistent with Andersen and Newman’s healthcare utilization framework, which views healthcare behavior as multidimensional and shaped by individual, healthcare system, and societal determinants (24). Some determinants—such as spiritual interpretation of illness, trust in TCM, administrative invisibility, and informal donor dependence—cannot be adequately captured through questionnaire scores alone. For this reason, the qualitative phase was essential, not supplementary. It provided the mechanism behind the statistical pattern.

The combined quantitative and qualitative findings show that healthcare inequity among aged Han Buddhist monks is produced through a dual process. On one side, religious values can provide meaning, resilience, and culturally legitimate forms of care. On the other side, these same values can constrain biomedical engagement when medical procedures are perceived as spiritually disruptive. In the same way, the monastery has the ability to protect the aging monks through internal solidarity, but can also be responsible for reproducing inequality when its capacity is inadequate. As such, older monks must be understood not only as the powerless who fall victim to healthcare discrimination. These are religious actors who face diseases using their values, disciplines within Buddhism, and available social support systems. However, in doing so, they act under the constraints of geographical, insurance, policy, and institutional inequalities. This dual perspective is critical in comprehending healthcare disparities among older religious people.

These findings have direct implications for policy and practice. First, healthcare programs for aged monks should use culturally sensitive health education rather than a purely biomedical communication model. Medical advice will be less effective if it dismisses TCM, meditation, or Buddhist meanings of suffering. The second recommendation is to integrate monasteries within community-based health systems by conducting regular health checks, using mobile clinics, keeping health records, referring patients, and training health coordinators within the monasteries. Thirdly, there needs to be a mechanism by which Buddhist groups and governments provide aid to rural and independent monasteries since this is where instability of donors and lack of access to healthcare are common. Fourth, healthcare professionals working with monks should be trained in Buddhist-sensitive communication. This includes acknowledging monks’ use of TCM, meditation, and spiritual practice; explaining biomedical treatment in non-coercive language; avoiding dismissive judgments toward religious healing preferences; and framing preventive care, chronic disease management, and necessary medical procedures as ways to preserve bodily wellbeing, reduce suffering, and support the continuity of monastic practice (20, 33, 36).

This study has several limitations. First, the quantitative sample was drawn from selected monasteries in Shandong and Zhejiang, so the findings cannot be generalized to all Han Buddhist monks in China. Second, the study relied in part on self-reported data, which may introduce recall bias, particularly when reporting healthcare access, treatment preferences, and perceived barriers. Third, the regression model included key variables related to religion, monastic support, and socio-economic background, but other possible determinants—such as chronic disease severity, exact insurance status, distance to the nearest hospital, and digital health literacy—were not fully modeled. Although the model shows that religious factors, monastic/cultural support, and socioeconomic background are significantly associated with healthcare access, it cannot fully capture the reciprocal and interdependent relationships among these factors. Future studies should consider longitudinal designs or more complex analytic approaches, such as mediation, moderation, or structural equation modeling, to examine how religious beliefs, monastic support, socioeconomic position, and healthcare access influence one another over time. Fourth, the qualitative sample captured three stakeholder groups, but did not include physicians or local primary healthcare workers who directly interact with monasteries. Future research should expand the sample across more provinces, include objective healthcare utilization data, compare different Buddhist traditions or monastery types, and examine how monastery-based health intervention models can reduce inequity in religious aging populations. There is another limitation related to the measure of education. In the present study, education was measured in terms of formal secular education and not in terms of Buddhist monastic education, scriptural knowledge, meditative education, ethical knowledge, or doctrinal knowledge acquired throughout one’s life time as individual variables. Furthermore, since formal education was considered within the socioeconomic background composite variable along with the other two variables, namely location of monastery and length of stay in monastery, the regression analysis cannot reveal the independent contribution of formal education to healthcare access.

## Conclusion

This research concludes that inequality in access to health services among aging Han Buddhist monks in China is shaped by the interaction between religious life, monastic support, and socio-economic position. Quantitative results show that religious factors hurt access to health services, while monastic/cultural support is the strongest positive predictor, followed by socio-economic background. Qualitative results suggest that preferences for TCM, meditation, and spiritual practices, as well as caution against invasive procedures, can delay monks’ engagement with biomedical care. On the contrary, the backing of co-monks, monastery officials, donors, Buddhist organizations, and governmental organizations may increase the availability of health care. The main barriers appear to have the strongest impact on rural and independent monasteries due to problems associated with transportations, costs, insurance, monastery health care, policy integration, and connections. In this regard, healthcare inequality among older Han Buddhist monks cannot simply be defined in terms of geography or economics. Instead, it is important to consider healthcare inequality among older Han Buddhist monks from a structural perspective based on religion, the capabilities of monasteries, and connections with the modern health care system.

## Data Availability

The original contributions presented in the study are included in the article/supplementary material, further inquiries can be directed to the corresponding author.
